# Dietary Intervention of Benzoic Acid for Intestinal Health and Growth of Nursery Pigs

**DOI:** 10.3390/ani14162394

**Published:** 2024-08-18

**Authors:** Hyunjun Choi, Sung Woo Kim

**Affiliations:** Department of Animal Science, North Carolina State University, Raleigh, NC 27695, USA; hchoi25@ncsu.edu

**Keywords:** benzoic acid, organic acids, growth performance, intestinal health, swine

## Abstract

**Simple Summary:**

Benzoic acid has been supplemented in feeds to improve intestinal health and the growth rate of nursery pigs. Benzoic acid, a colorless crystalline organic compound, consists of a benzene ring with a carboxyl substituent, representing the simplest form of aromatic carboxylic acid. In pig feeds, benzoic acid primarily influences the small intestine rather than the large intestine of pigs, as benzoic acid does not require degradation in the stomach or beyond the small intestine. Benzoic acid possesses acidic properties, making it an effective feed additive in pig production through its antimicrobial action by reducing pH in feed and digesta in the gastrointestinal tract, positively modulating intestinal microbiota and enhancing nutrient utilization and intestinal health in pigs. Benzoic acid supplementation also decreases urinary pH through the production of hippuric acid and reduces ammonia emissions, which can positively affect the conditions of barns considering the intense pig production systems. However, depending on the levels of benzoic acid, its impact on growth is not always consistent. Thus, this review investigated the functional roles of benzoic acid on the intestinal health, nutrient utilization, and growth performance of nursery pigs, determined the optimal dose level of benzoic acid on the growth rate in nursery pigs, and compared the efficacy of benzoic acid and other acids, aiming to clarify the potential benefits of benzoic acid in pig feeds.

**Abstract:**

The objectives of this review are to investigate how benzoic acid can mitigate the negative effects of weaning stress, improve the intestinal microbiota, intestinal health, and growth of nursery pigs, determine the optimal dose level of benzoic acid for the growth rate in nursery pigs, and compare the efficacy of benzoic acid and other acids in pig feeds. After weaning, pigs are exposed to less lactose and solid feed with high acid-binding capacity at infrequent intervals, causing an increase in digesta pH, reducing protein digestion, and increasing ammonia-producing bacteria in the stomach. Benzoic acid supplementation has improved the intestinal health and growth of nursery pigs through its antimicrobial properties and pH reduction in the digesta. The positive modulation of luminal microbiota in the small intestine of pigs by benzoic acid improves intestinal morphology and enhances nutrient utilization, especially nitrogen, of nursery pigs. Benzoic acid supplementation of up to 1% in feeds also increases hippuric acid contents in the urine of nursery pigs, decreasing urinary pH, which is related to ammonia emission and barn conditions in intensive pig production. Supported by the beneficial impacts of benzoic acid, the growth performance of nursery pigs was also improved. However, excessive benzoic acid (over 2.5% up to 5%) in feeds reduces the growth performance of nursery pigs. Thus, this review conducted a meta-analysis of the results from 16 papers to determine the optimal dose level of benzoic acid for body weight gain of nursery pigs, which was found to be 0.60%. The efficacy of benzoic acid was similar to that of other organic acids, including citric acid, fumaric acid, formic acid, and formate salts. Collectively, benzoic acid supplementation can positively modulate the luminal and mucosal microbiota in the small intestine, increase nutrient utilization and intestinal health, decrease urinary pH, and improve the growth performance of nursery pigs.

## 1. Introduction

The weaning period is the most critical phase for pigs, marked by intestinal challenges, diarrhea, and growth reduction [1,2]. Newly weaned pigs face various stressors, making the small intestine highly susceptible to disruptions of the intestinal microbial ecosystem, inflammation, and oxidative stress, further reducing intestinal health and growth [3,4]. During the post-weaning period, weaning stress dramatically decreases the feed intake of pigs, limiting nutrients for bacterial survival and proliferation, reducing intestinal morphology, and leading to intestinal barrier dysfunction [5]. Furthermore, weaning stress negatively impacts microbiota establishment in the small intestine, decreasing the relative abundance of acid-producing bacteria and increasing that of ammonia-producing bacteria such as *Escherichia coli* [6]. The reduction of acid-producing bacteria raises the intestinal pH, increasing the possibility of disease susceptibility in the small intestine of pigs and inducing long-lasting effects on the intestinal health and growth of pigs. Antibiotics have been commonly used in nursery feeds to mitigate the negative impacts of weaning stress and improve the intestinal health and growth of pigs [7,8]. However, concerns about microbial resistance have led to the phasing out of antibiotics in animal feeds in many countries [9,10]. This has created a need for antibiotic alternatives in pig production to positively modulate the intestinal microbiota, to enhance the intestinal health and growth of pigs [11]. Among these alternatives, benzoic acid has gained attention for its antimicrobial properties and positive effects on intestinal microbiota, intestinal health, nutrient utilization, and the growth of pigs [12,13].

Benzoic acid, a colorless crystalline organic compound, consists of a benzene ring with a carboxyl substituent, representing the simplest form of aromatic carboxylic acid. Benzoic acid has shown a positive impact on the modulation of microbiota in the small intestine of nursery pigs, increasing beneficial bacteria such as *Lactobacillus* spp. and decreasing *Escherichia coli* [12,14]. Benzoic acid also positively influenced intestinal morphology and nutrient utilization of nursery pigs [15]. Benzoic acid supplementation in feeds increased benzoic acid and hippuric acid contents in urine with a decrease in the urinary pH of pigs [16], which is highly related to ammonia emission and conditions of the barn in intensive swine production [17]. Supported by the beneficial impacts of benzoic acid, the growth performance of pigs was improved [18,19,20]. However, the supplementation of over 2.5% up to 5% benzoic acid supplementation decreased the growth performance of nursery pigs [21], requiring a clear understanding of the antimicrobial properties of benzoic acid for the modulation of intestinal microbiota and the enhancement of intestinal health and growth. Therefore, the objectives of this review are to investigate the functional roles of benzoic acid in the intestinal microbiota, intestinal health, nutrient utilization, and growth of nursery pigs, determine the optimal dose level of benzoic acid for body weight gain of nursery pigs, and compare the efficacy of benzoic acid and other acids in pig feeds.

## 2. The Importance of Dietary Intervention for Intestinal Health and Growth of Nursery Pigs

Weaning is the most critical event in the life of a pig, characterized by disruptions in the intestinal microbial ecosystem, intestinal challenges, diarrhea, and growth reduction [1,2]. Weaned pigs are challenged by solid feeds that contain antinutritional, allergenic, and antigenic compounds, leading to feed-intake reduction, causing intestinal inflammation, and negatively affecting intestinal morphology and growth [2]. During the post-weaning period, the reduced feed intake of pigs also limits the nutrients available for bacterial survival and proliferation, negatively affecting the intestinal microbiota and intestinal health [1,6].

The intestinal microbiota plays a crucial role in maintaining the intestinal health and growth of pigs, leading to increased attention to the interaction between intestinal microbiota and intestinal health [4,22]. The beneficial bacteria in the intestine of pigs prevent the colonization of opportunistic pathogenic or ammonia-producing bacteria and reduce the risk of inflammation in the small intestine of pigs [23]. On the other hand, ammonia-producing bacteria that cause bacterial infections, such as F18+ *Escherichia coli*, negatively modulate the jejunal microbiota, increase intestinal permeability in the jejunum, induce inflammation, and reduce intestinal morphology and growth performance of nursery pigs [8]. Additionally, the luminal and mucosa-associated microbiota in the jejunum contribute to the maintenance of the intestinal barrier function and the prevention of the translocation of harmful pathogens and toxins into pigs [5]. Thus, weaning stress can heavily influence the luminal and mucosal microbiota, which can be a main contributor to the reduction of intestinal health and growth performance of nursery pigs.

The microbiota is highly dependent on the nutrients in the digesta of pigs, indicating that the diet being fed and ingested has a major influence on the intestinal microbiota [23]. The small intestine of nursery pigs faces these challenges directly as it constitutes the largest absorptive surface in the gastrointestinal tract, coming into close contact with dietary compounds, the microbiota, and exogenous toxins [4]. The presence of an abundance of immune cells and their interaction with the microbiota in the small intestine indicates that the small intestine is susceptible to dietary compounds [24,25]. The mucosa-associated microbiota in the small intestine of pigs plays a crucial role in interacting with intestinal immune cells and serves as the primary defense against opportunistic pathogens [26]. The immune cells, including dendritic cells and microfold cells in the enterocyte, sense intestinal antigens mainly through toll-like receptors and nucleotide-binding oligomerization domain-like receptors to modulate cytokine production for the maintenance of immune functions and intestinal health in pigs [23]. The increased presence of cytokines induces an increased inflammatory process and increased oxidative damage products in the jejunum of pigs. The increased inflammation and oxidative stress in the jejunum lead to decreased intestinal morphology, increased gut permeability, and increased energy costs required for the recovery of intestinal barrier functions, all of which compromise the growth of pigs [6]. Therefore, dietary intervention to positively modulate luminal and mucosa-associated microbiota in the jejunum becomes a critical factor for intestinal health, nutrient utilization, and growth of nursery pigs.

## 3. Exploring Dietary Intervention of Benzoic Acid on Intestinal Microbiota, Intestinal Health, and Growth of Nursery Pigs

### 3.1. Characteristic and Mechanism of Action of Benzoic Acid in Pigs

Benzoic acid possesses acidic properties, making it an antibiotic alternative in pig feeds due to its antimicrobial action through pH reduction in feeds and digesta [27,28]. Benzoic acid has been supplemented in food as a preservative for several decades, and benzoic acid has been registered as a feed additive for pig feeds in many countries. Benzoic acid in feeds is mainly absorbed rapidly and does not require degradation in the stomach or later than in the small intestine of pigs [29].

**Figure 1:**
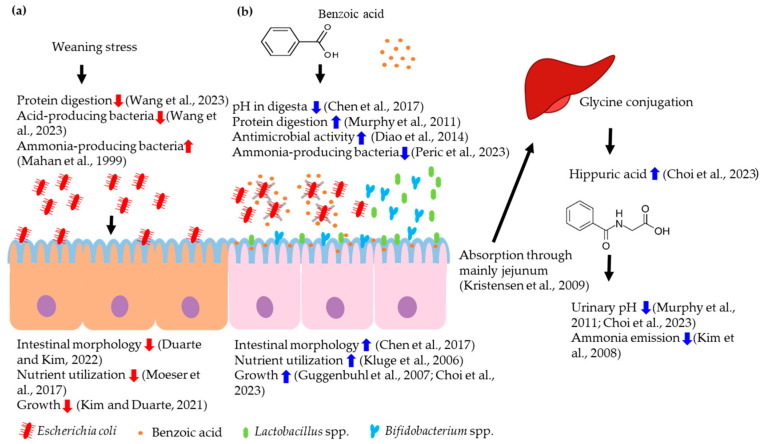
(**a**) Negative effects of weaning stress on intestinal health and growth of nursery pigs [4,5,23,31,32]; (**b**) mechanism of benzoic acid on luminal microbiota, intestinal health, nutrient utilization, urinary pH, and growth performance of nursery pigs [12,13,14,16,18,27,28,29,33,34,35,36]. Red arrows indicate effects of weaning stress and blue arrows indicate effects of benzoic acid.

After absorption in the small intestine, benzoic acid can serve as an additional energy source for pigs. However, the additional energy effects in pig nutrition may not be the main impact on the intestinal health and growth of pigs due to the low inclusion rate in feeds. Importantly, the benzoic acid has antimicrobial properties, making it an antibiotic alternative in pigs [19]. Thus, the main proposed modes of action of benzoic acid in pig feeds are (1) antimicrobial effects for intestinal health, (2) the improvement of nutrient utilization, and (3) the reduction of feedborne pathogens via pH reduction in digesta (Figure 1). Benzoic acid exerts antimicrobial effects primarily through its mode of action, with the main factor being its pKa value, which represents the acid dissociation constant. A greater pKa indicates a lesser extent of acid dissociation. The antimicrobial effectiveness of organic acids depends on the ability to transition between associated and dissociated forms based on the environmental pH. This property enables them to effectively penetrate the semipermeable membrane of bacteria when in the associated form. Once benzoic acid is inside the cell, where the pH is typically around 7, the benzoic acid dissociates and inhibits essential cell enzymes such as decarboxylase and catalase- as well as nutrient-transport systems. The efficacy of an acid in microbial inhibition is closely dependent on the pKa value of organic acid, which represents the pH at which 50% of the acid is dissociated. Organic acids with higher pK values serve as more effective preservatives, and their antimicrobial efficacy tends to improve with increasing chain length and degree of unsaturation [30]. Benzoic acid can also be supplemented either as free acids or as salts [13], with the latter having the advantages of being less corrosive, less odorous, and easier to handle compared with free acids. Another relatively new form used in swine nutrition is coated acids.

### 3.2. Lowering Digesta pH in Pigs

High lactose consumption from milk in suckling piglets increases lactic acid content in the stomach, creating a sufficiently acidic condition that delays the proliferation of enterotoxigenic *Escherichia coli*, even though the pigs do not have the ability to produce sufficient hydrochloric acid [37]. However, after weaning pigs are exposed to less lactose and solid feed with a high acid-binding capacity at infrequent intervals, causing an increase in digesta pH, a reduction in protein digestion, and an increase in ammonia-producing bacteria in their stomach [31]. Acid-binding capacity in feeds represents the resistance of a feedstuff to pH reduction by gastric acid [38]. The problem is that feedstuffs commonly used for nursery pigs, especially mineral supplements, have a high acid-binding capacity [39], which can decrease protein digestion and reduce the average daily gain (ADG) of nursery pigs [31]. The insufficient acidity in the stomach of nursery pigs also lasts for 3 weeks, which causes reduced protein digestion, and pigs become susceptible to ammonia-producing and opportunistic pathogenic bacteria attachment in the stomach [32]. Digesta pH in the stomach of pigs is a critical factor for protein digestion as it influences proteolytic enzyme efficiency and protein solubility [40]. Protein digestion starts in the stomach with pepsin, activated from pepsinogen by hydrochloric acid. The conversion to pepsin is increased in lower pH, around 2.0, compared to high pH, indicating that acidic conditions in the stomach increase efficiency in protein digestion. However, some previous studies reported that organic acid supplementation has a relatively positive impact on protein digestion during the early age period (the 1st and 2nd week after weaning) compared to the older age [41,42], indicating that the effects of benzoic acid on digesta acidification may occur in the early age of pigs (Table 1). However, the pathogenic bacteria attachment in the gastrointestinal tract in newly weaned pigs has long-lasting effects on intestinal health and growth [43]. Additionally, a previous study has demonstrated that the supplementation of benzoic acid reduces digesta pH in the stomach of pigs, decreases pathogenic bacteria attachment in the small intestine, and thereby improves intestinal health and growth performance throughout the overall nursery phase [33] (Table 2).

### 3.3. Modulation of Intestinal Microbiota and Positive Impact on Intestinal Health, Nutrient Utilization, and Growth of Pigs

**Figure 2:**
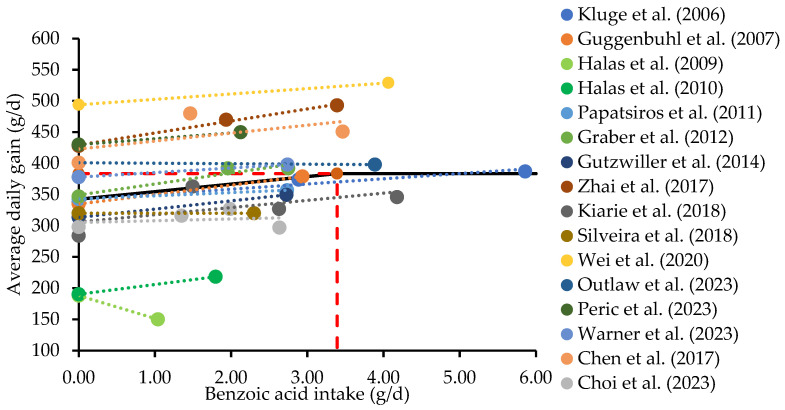
Average daily gain of nursery pigs fed diets with various benzoic acid intake. The meta-analysis is conducted by Proc NLMIXED using the data from 16 peer-reviewed papers to determine a breakpoint when an increase of average daily gain is plateaued [13,14,15,18,19,27,28,33,35,44,56,57,58,59,60,61]. The breakpoint (a one-slope broken-line analysis) was 3.39 g (standard error = 1.57; *p* < 0.05) of daily benzoic acid intake by nursery pigs. The equation was: ADG (g/d) = 383.6 − 12.09 × z1 (benzoic acid intake, g/d), R^2^ = 0.95 if benzoic acid intake is ≥breakpoint, then z1 = 0. The optimal dose level (0.60%) of benzoic acid for average daily gain was calculated based on average daily feed intake (0.566 kg/d).

The interaction between the intestinal microbiota and intestinal health has gained attention for the improvement of pig production [23]. Modulation of the intestinal microbiota significantly influences intestinal health and the growth of nursery pigs due to long-lasting effects on the early intestinal development of intestinal function and the immune system [22,43]. Previous studies have shown that weaning negatively impacts the establishment of microbiota in the small intestine of pigs, decreasing the relative abundance of acid-producing bacteria and increasing that of ammonia-producing bacteria such as *Escherichia coli* [6]. The post-weaning period in pigs is also susceptible to *Escherichia coli* infections, increasing the potential to induce inflammatory responses, increase oxidative damage products, disrupt the barrier function in the small intestine, and consequently compromise intestinal morphology and the growth of pigs [8,53]. Benzoic acid supplementation has improved intestinal health and the nutrient utilization of nursery pigs (Table 3). However, a previous study reported that over 2.5% and 5% benzoic acid supplementation in feeds decreased the growth performance of nursery pigs [41]. The possible reason for the observed decrease in growth performance is due to increased systemic acidosis in pigs, negatively affecting growth performance. Excessive benzoic acid supplementation of over 2.5% in diets impairs the functions of red and white blood cells, reducing their abilities of oxygen transport and immune status, and increasing oxidative stress in the hepatic cells of pigs [21]. The high levels of benzoic acid also reduce glycine availability for the growth of pigs due to the increased use of protein and amino acids for the conversion to hippuric acid in the liver [47]. Another possibility for the reduced growth performance of pigs may be that high levels of organic acids reduce palatability [54,55], which agrees with a previous study of pigs fed over 2.5% benzoic acid in feeds [21]. However, 1% benzoic acid supplementation in feeds did not reduce the feed intake of nursery pigs [27]. Thus, a meta-analysis was conducted to determine the optimal dose level of benzoic acid in feeds for body weight gain using results from 16 peer-reviewed articles. A literature search was conducted using the database in PubMed and Google Scholar with keywords including benzoic acid, organic acids, intestinal health, growth performance, and pigs, followed by screening after reading each article. During this screening process, only the articles showing the growth performance of the entire nursery period of pigs were selected. The initial body weight and experimental period ranges for the nursery phase were 4.7 to 9.7 kg and 21 to 42 days, respectively (Table 2). The benzoic acid intake in each research article was calculated by multiplying benzoic acid content with the overall average daily feed intake of each treatment. The benzoic acid used was in free form except for two articles [14,56] where coated forms were used. Diets used in these articles were largely based on corn–soybean meal (8 articles). Based on the meta-analysis, the optimal dose of benzoic acid for the average daily gain of nursery pigs was 0.60%, which can improve ADG by around 10% compared with the control group (Figure 2).

**Table 2:** Growth performance of nursery pigs fed diets with various benzoic acid (BA) contents ^1^.

IBW ^2^ (kg)	Experimental Period (d)	BA (%)	Growth Performance ^3^ (% Change ^4^)			Reference
			ADG	ADFI	G:F	
7.3	35	0.50	10.7	8.5	2.0	[27]
		1.00	14.5 **	10.2	3.9	
7.4	32	0.50	13.1 **	6.3	6.5 **	[35]
5.0	21	0.50	−20.2	−17.5	−3.3	[15]
6.0	21	0.50	14.7 **	10.9 **	3.4	[18]
7.9	35	0.50	4.1 **	2.6 **	1.4	[57]
6.5	42	0.35	13.0 **	10.9 **	1.9	[28]
		0.50	13.0 **	9.1 **	3.5	
9.7	42	0.50	11.5 *	13.2 **	−1.5	[44]
6.7	42	0.20	19.7 **	13.7 **	5.3 **	[33]
		0.50	12.5	7.6 **	4.5 **	
7.1 ^5^	28	0.30	9.8	5.9	3.7	[58]
		0.50	15.2	11.3	3.5	
6.2	42	0.50	7.1	0.4	6.7 **	[59]
6.4 ^6^	42	0.25	27.8	17.7	8.6	[19]
		0.50	15.1	3.5	11.2	
		0.75	21.8	9.6	11.1	
4.7	40	0.50	0.0	−4.2	4.6	[60]
8.7	21	0.60	−0.7	5.9	−6.3	[56]
6.8	42	0.25	4.7	−2.3	7.1 **	[14]
6.2	38	0.50	5.3 **	4.0 **	1.3 *	[61]
6.9 ^7^	41	0.25	6.0	5.1	0.9	[13]
		0.35	9.7	10.3	−0.5	
		0.50	−0.3	2.5	−2.8	
	Average		9.5	6.1	3.2	

^1^ Asterisk marks (*, **) represent statistical tendency (*p* < 0.10) and difference (*p* < 0.05), respectively. ^2^ IBW = initial body weight. ^3^ ADG = average daily gain, ADFI = average daily feed intake, G:F = gain-to-feed ratio. ^4^ The percentage increase or decrease in the ADG, ADFI, and G:F was determined in benzoic acid supplementation groups related to control group. ^5^ Increasing levels of BA linearly increased (*p* < 0.05) the ADG and ADFI. ^6^ Increasing levels of BA linearly increased (*p* < 0.05) the ADG and G:F. ^7^ Increasing levels of BA tended to quadratically increase (*p* < 0.05) the ADG.

The meta-analysis is conducted by Proc NLMIXED of SAS (SAS Inst. Inc., Cary, NC, USA) using the data from 16 peer-reviewed papers to determine a breakpoint when an increase of average daily gain is plateaued [13,15,18,19,27,28,33,35,44,56,57,58,59,60,61]. The breakpoint (a one-slope broken-line analysis) was 3.39 g (standard error = 1.57; *p* < 0.05) of daily benzoic acid intake by nursery pigs. The equation was: ADG (g/d) = 383.6 − 12.09 × z1 (benzoic acid intake, g/d), R^2^ = 0.95 if benzoic acid intake is ≥breakpoint, then z1 = 0. The optimal dose level (0.60%) of benzoic acid for the average daily gain was calculated based on an average daily feed intake (0.566 kg/d). The statistical significance and tendency were declared at *p* < 0.05 and 0.05 ≤ *p* < 0.10, respectively.

The proposed mode of action for improved growth performance involves benzoic acid’s antimicrobial effects on intestinal bacteria and its role in preventing diarrhea, thereby promoting intestinal health [19]. With a pKa value of 4.2, benzoic acid is relatively less acidic compared with other organic acids, but it still demonstrates potential for modulating intestinal bacteria in pigs [12]. Benzoic acid penetrates bacterial membranes, making an acidic condition within the bacteria that inhibits the proliferation of pathogenic bacteria strains through the H+-ATPase [29]. Dissociated benzoic acids within the cell release charged anions and protons, causing membrane disruption of bacteria, ion accumulation, an alteration of intracellular pH homeostasis, and, thereby, the suppression of metabolic activity in the Krebs cycle [62]. Organic acids and their derivatives exhibit antimicrobial effects against both gram-positive and gram-negative bacteria, with acid forms having stronger activities than ester forms [63]. However, structural differences between the gram-positive and gram-negative bacteria exist, creating different membrane permeability by acidic compounds [64]. Associated and uncharged organic acids can penetrate the cell membrane of bacteria as the organic acids have lipophilic conditions [65]. Gram-positive bacteria have a thick layer of peptidoglycan in their cell wall, which allows for a gram stain in the crystal violet dye [66], whereas the gram-negative bacteria have a relatively thinner layer of peptidoglycan in the cell walls with lipopolysaccharides [67]. The lipopolysaccharides in the outer membrane of gram-negative bacteria act as an additional barrier, making an attachment by acidic compounds more difficult compared with the gram-positive bacteria. Additionally, the correlation between pathogenic bacteria inhibition is highly correlated with sources of organic acids rather than pH level in vitro, possibly indicating that the source of organic acid is critical to modulate the intestinal microbiota, intestinal health, and growth of pigs [63]. In pigs, benzoic acid is primarily absorbed and transported in the jejunum through monocarboxylic acid transporters [29]. These facts indicate that before absorption, benzoic acid mainly influences the intestinal bacteria in the small intestine of nursery pigs. Furthermore, benzoic acid supplementation decreased gram-negative bacteria such as *Escherichia coli* in the duodenum of nursery pigs [27], increased *Lactobacillus* and *Bacillus* populations in the jejunal digestive contents [33], and increased the diversity of microbiota in the small intestine of nursery pigs [68], which positively influenced the intestinal health and growth of pigs. Lastly, the inhibitory effects of benzoic acid on pathogenic microorganisms make it a valuable preservative in both food and feed [69].

Benzoic acid supplementation also improves nutrient utilization in pigs, especially nitrogen [28,34,48,60]. The fewer protein contents in the digesta in the gastrointestinal tract decreased the possibility of ammonia-producing bacteria populations and decreased the incidence of post-weaning diarrhea in nursery pigs [70]. The benzoic acid supplementation also decreased ammonia-nitrogen and increased short-chain fatty acid contents in the gastrointestinal tract of nursery pigs [12]. This benzoic acid effect can be attributed to increased positive modulation of intestinal bacteria, digestive enzyme activities [33], and improvements in intestinal morphology [12,14]. Benzoic acid supplementation upregulates the expression of the glucagon-like peptide 2 gene [45], insulin-like growth factor 1, insulin-like growth factor 1 receptor, and tight junction proteins including occludin and zonula occludens 1 in the jejunum of nursery pigs [33]. The improved intestinal morphology in the jejunum is highly associated with acidic conditions in the intestine, increased enzymatic activity in the brush border of enterocytes, increased short-chain fatty acid production, and upregulated proteins related to intestinal development [23]. Based on the literature, benzoic acid supplementation in feeds at 0.25 to 0.50% positively modulates luminal microbiota by increasing beneficial bacteria such as *Lactobacillus* spp., decreasing *Escherichia coli*, and increasing intestinal morphology (Table 3), collectively improving growth performance. Besides the antimicrobial effects of benzoic acid, benzoic acid decreased urine pH in pigs [13].

Once benzoic acid is absorbed, benzoic acid undergoes metabolism in the liver, where benzoic acid conjugates with glycine to form hippuric acids, which are then excreted through urine [29]. Based on the literature regarding the effects of benzoic acid on urinary pH changes in pigs, benzoic acid supplementation up to 1% in nursery pigs or 3% in growing pigs decreased urinary pH (Table 2), indicating that benzoic acid is excreted through urine in pigs. The urinary pH reduction is mainly from hippuric acid, which decreases ammonia emission [16,36]. The reduced ammonia emission by benzoic acid also positively impacts barn conditions considering the intense pig-production system [17].

In addition to benzoic acid, many types of organic acids with antimicrobial properties have been widely used [41,42]. In this review, benzoic acid supplementation to nursery feed increased the average daily gain (ADG) by approximately 9.5% (Table 2). During the overall nursery phase, citric acid increased ADG by 3%, fumaric acid by 5%, formic acid by 7.1%, and formate salts by 6.9% compared with no-organic-acid diets (Table 4). Thus, based on the efficacy of organic acids, benzoic acid had similar impacts with other organic acids, including citric acid, fumaric acid, formic acid, and formate salts.

**Table 3:** Intestinal microbiota, intestinal health, and nutrient utilization of pigs fed diets with various benzoic acid (BA) contents.

Item	IBW ^1^ (kg) or Age (d)	Experimental Period (d)	BA (%)	Results ^2^	Reference
Luminal microbiota	7.3 kg	35	1.00	Decreased gram-negative bacteria in duodenum and total aerobic bacteria in ileum	[27]
	6.8 kg	42	0.25	Decreased *Enterococcus* spp. in duodenum, decreased *Escherichia coli* in jejunum, increased *Clostridium perfringens* in ileum, increased *Lactobacillus* spp. in ileum, and increrased *Lactobacillus* spp. in cecum	[14]
	7.4 kg	32	0.50	Decreased total lactic acid bacteria in the stomach, decreased *Escherichia coli* in cecum	[35]
	8.9 kg	28	0.50	Increased biodiversity in ileum	[68]
	6.0 kg	14 and 42	0.50	Increased *Bifidobacterium* and decreased *Escherichia coli* in ileum on d 14 tended to decrease *Escherichia coli* in cecum on d 14, increased *Bacillus* and decreased *Escherichia coli* in the ileum on d 42 and decreased *Escherichia coli* in the cecum on d 42	[12]
	6.5 kg	14 and 42	0.20	Increased *Bacillus* in the ileal digesta, decreased *Escherichia coli* in the cecal digesta on d 14, increased *Lactobacillus*, *Bifidobacterium*, decreased *Escherichia coli* in the ileal digesta on d 42, increased *Bifidobacterium*, decreased *Escherichia coli* in the cecal digesta on d 42	[33]
			0.50	Increased *Lactobacillus* and *Bacillus* in the ileal digesta on d 14, decreased *Escherichia coli* in the ileal digesta on d 42, decreased *Escherichia coli* in the cecal digesta on d 42	[33]
Intestinal health and nutrient utilization	6.8 kg	42	0.25	Increased villus height, decreased crypt depth, and increased VH:CD in the small intestine, including duodenum and jejunum	[14]
	6.0 kg	42	0.50	Increased VH:CD in the small intestine, including duodenum, jejunum, and ileum on d 14 and d 42	[12]
	5.9 kg	21	0.50	Increased villus height in the ileum and increased AID of CP	[18]
	7.3 kg	35	0.50	Increased N retention	[27]
	6.2 kg	42	0.50	Increased ATTD of CP	[59]
	7.4 kg	32	0.50	Increased ATTD of CP and energy	[35]
	6.5 kg	42	0.50	Increased N retention	[28]
	6.5 kg	14 and 42	0.20	Increased villus height, decreased crypt depth, and increased VH:CD in the jejunum on d 14 and increased villus height and VH:CD in the jejunum on d 42	[33]
			0.50	Decreased crypt depth, increased VH:CD in the jejunum on d 14, and increased VH:CD in the jejunum on d 42	
	21 d	35	0.50 and 1.00	Increased ATTD of N	[34]
	18.8 kg	14	0.50	Decreased crypt depth, increased VH:CD in the jejunum, trypsin, lipase, and amylase activities in the jejunum, and increased ATTD of CP, dry matter, ether extract, energy, and ash	[12]
	33.1 kg	53	0.50	Increased N retention	[48]
	26.0 kg	84	1.00	Increased ATTD of N	[47]
	64.0 kg	14	1.00, 2.00, and 3.00	Linearly decreased total N excretion and tended to linearly increase N retained/intake	[16]
	45.0 kg	5	2.00	Increased ATTD of Ca and P	[50]
	29.9 kg	6	1.00	Increased retained N of absorbed (%)	[52]

^1^ IBW = initial body weight. ^2^ VH:CD = villus height to crypt depth ratio, AID = apparent ileal digestibility, ATTD = apparent total tract digestibility, CP = crude protein, N = nitrogen.

**Table 4:** Efficacy of benzoic acid and other acids on average daily gain (ADG) in nursery pigs.

Item	IBW ^1^ (kg)	Inclusion Rate (%)	ADG ^2^ (% Changes)		No. Exp	Reference
			Average	Range		
Benzoic acid	5.0 to 9.7	0.20 to 1.00	9.5	−20.2 to 27.8	16	Table 2
Citric acid	4.2 to 9.6	0.50 to 3.00	3.0	−8.1 to 12.2	12	[55,71,72,73,74,75,76,77,78,79,80]
Fumaric acid	5.7 to 9.6	0.20 to 4.00	5.0	−11.8 to 21.1	15	[38,55,71,72,73,76,79,81,82,83,84]
Formic acid	5.7 to 9.1	0.20 to 2.40	7.1	−15.1 to 26.9	7	[54,78,81,84,85,86,87,88]
Formate salts	5.6 to 10.0	0.40 to 2.80	6.9	−9.4 to 22.9	9	[27,68,89,90,91]

^1^ IBW = initial body weight. ^2^ The percentage increase or decrease in the ADG of pigs was determined in acid supplementation groups related to control group.

## 4. Conclusions

The current review of the available research shows that benzoic acid has positive impacts on the modulation of luminal microbiota in the small intestine of nursery pigs, increasing beneficial bacteria such as *Lactobacillus* spp. and decreasing *Escherichia coli*, mainly through antimicrobial properties and pH reduction in the digesta. These microbial changes in the lumen of the small intestine positively influence intestinal morphology and nutrient utilization by nursery pigs, resulting in improved growth performance. Benzoic acid supplementation of up to 1% in feeds increases urinary hippuric acid, decreasing urine pH and thus reducing ammonia emission from pig manure. However, excessive benzoic acid supplementation (over 2.5% up to 5%) could negatively affect the growth performance of nursery pigs due to increased acidosis and reduced glycine availability in the body. A meta-analysis of the results from 16 peer-reviewed papers showed the optimal dose level of benzoic acid for weight gain of nursery pigs at 0.60%. Benzoic acid has a similar efficacy compared with other organic acids, including citric acid, fumaric acid, formic acid, and formate salts for its antimicrobial properties, as well as improvements to intestinal health and growth performance of nursery pigs.

## Figures and Tables

**Table 1:** Digesta pH, urinary pH, hippuric acid, and benzoic acid contents in urine of pigs fed diets with various benzoic acid (BA) contents ^1^.

IBW ^2^ (kg) or Age (d)	Experimental Period (d)	BA (%)	Acidification (% Change ^3^)					
			pH in Stomach	pH in Jejunum	pH in Urine	HA ^4^ in Urine	BA in Urine	Reference
6.5 kg	42	0.35	-	-	−7.98 **	681.1 **	−35.14	[28]
		0.50	-	-	−10.24 **	745.3 **	86.49	
7.3 kg	35	0.50	−4.32	−0.65	−7.79	-	-	[27]
		1.00	−4.32	0.16	−13.83 **	-	-	
6.8 kg	42	0.25	−15.63	−7.12 **	-	-	-	[14]
9.7 kg	42	0.50	-	-	−8.83 **	-	-	[44]
6.0 kg	14	0.50	-	−6.93	-	-	-	[12]
6.0 kg	42	0.50	-	−5.47	-	-	-	[12]
6.5 kg	14	0.20	−10.15	−4.23 **	-	-	-	[33]
		0.50	−17.54 **	−5.48 **	-	-	-	
6.5 kg	42	0.20	−10.59	−1.25	-	-	-	[33]
		0.50	−26.61 **	−8.31	-	-	-	
18.8 kg	14	0.50	-	−8.06 *	-	-	-	[45]
6.6 kg	35	0.30	−7.93	-	−6.42 **	-	-	[46]
		0.50	−26.90	-	−3.83 **	-	-	
21 d	35	1.00	-	-	−15.74 **	-	-	[36]
Nursery phase (average):			−13.78	−4.73	−9.33	713.20	25.68	
26.0 kg	42	1.00	-	-	−11.78 **	886.3 **	-	[47]
26.0 kg	84	1.00	-	-	−12.32 **	1019.5 **	-	[47]
33.1 kg	53	0.50	-	-	−5.56 **	-	-	[48]
63.0 kg	N/A ^7^	1.00	-	-	−28.82 **	2064.5 **	752.33 **	[29]
28.0 kg ^5,6^	21	1.00	-	-	−10.52	-	-	[49]
		2.00	-	-	−27.32	-	-	
64.0 kg ^5^	14	1.00	-	-	−12.21	-	-	[16]
		2.00	-	-	−29.58	-	-	
		3.00	-	-	−32.28	-	-	
45.0 kg	5	2.00	-	-	−11.21 **	612.9 **	4868.75 **	[50]
50.0 kg	10	1.00	-	-	−12.14 **	1500.0 **	1341.18 **	[51]
		2.00	-	-	−24.56 **	2733.3 **	2594.12 **	
29.9 kg	6	1.00	-	-	−12.35 **	1858.4 **	-	[52]
Growing phase (average):			-	-	−17.74	1525.0	2389.1	

^1^ Asterisk marks (*, **) represent statistical tendency (*p* < 0.10) and difference (*p* < 0.05), respectively. ^2^ IBW = initial body weight. ^3^ The percentage increase or decrease in pH in the stomach, jejunum, and urine, hippuric acid in urine, and benzoic acid in urine was determined in benzoic acid supplementation groups related to control group. ^4^ HA = hippuric acid. ^5^ Increasing levels of BA linearly decreased (*p* < 0.05) urinary pH. ^6^ Increasing levels of BA quadratically decreased (*p* < 0.05) urinary pH. ^7^ N/A = not available.
